# Congenital hyperinsulinism: clinical and molecular characterisation of compound heterozygous *ABCC8* mutation responsive to Diazoxide therapy

**DOI:** 10.1186/1687-9856-2014-24

**Published:** 2014-12-15

**Authors:** Ved Bhushan Arya, Qadeer Aziz, Azizun Nessa, Andrew Tinker, Khalid Hussain

**Affiliations:** London centre for Paediatric Endocrinology, Great Ormond Street Hospital for Children NHS Foundation Trust, London WC1N 3JH and The Institute of Child Health, University College London, London, WC1N 1EH UK; The Heart Centre, William Harvey Research Institute, Barts and the London School of Medicine and Dentistry, Charterhouse Square, London, UK; The Institute of Child Health, University College London, London, WC1N 1EH UK; Developmental Endocrinology Research Group, Molecular Genetics Unit, Institute of Child Health, University College London, 30 Guilford Street, London, WC1N 1EH UK

**Keywords:** Congenital hyperinsulinism, Hypoglycaemia, *ABCC8*, Diazoxide

## Abstract

**Background:**

Mutations in *ABCC8* and *KCNJ11* are the most common cause of congenital hyperinsulinism (CHI). Recessive as well as dominant acting *ABCC8/KCNJ11* mutations have been described. Diazoxide, which is the first line medication for CHI, is usually ineffective in recessive *ABCC8* mutations. We describe the clinical and molecular characterisation of a recessive *ABCC8* mutation in a CHI patient that is diazoxide response.

**Clinical case:**

A term macrosomic female infant presented with symptomatic persistent hypoglycaemia confirmed to be secondary to CHI. She exhibited an excellent response to moderate doses of diazoxide (10 mg/kg/day). Molecular genetic analysis of the proband confirmed a biallelic *ABCC8* mutation – missense R526C inherited from an unaffected mother and a frameshift c.1879delC mutation (H627Mfs*20) inherited from an unaffected father. Follow-up highlighted persistent requirement for diazoxide to control CHI. Functional analysis of mutants confirmed them to result in diazoxide-responsive CHI, consistent with the clinical phenotype.

**Conclusion:**

Biallelic *ABCC8* mutations may result in diazoxide-responsive CHI. Irrespective of the molecular genetic analysis results, accurate assessment of the response to diazoxide should be undertaken before classifying a patient as diazoxide-responsive or unresponsive CHI.

## Background

Congenital hyperinsulinism (CHI) is due to an inappropriate insulin secretion by the β-cells of the islets of Langerhans [1]. It usually presents with severe hypoketotic hypofattyacidaemic hypoglycaemia [2]. The majority of the affected newborns are macrosomic at birth and require high intravenous glucose administration to maintain plasma glucose above 3.5 mmol/l [3].

Mutations in *ABCC8* and *KCNJ11*, which encode the SUR1 and Kir6.2 subunits of pancreatic ATP-sensitive potassium channel (K_ATP_), are by far the most common cause of CHI and are estimated to account for 36%-69% of all cases [4–6]. K_ATP_ channels are octameric protein complexes composed of four pore-forming Kir6.2 subunits and four sulfonylurea receptor 1 (SUR1) subunits, and form a link between cellular metabolism and membrane excitability [7, 8]. It is thought that the β-cells in patients with CHI are persistently depolarized because of abnormally modulated or absent K_ATP_ channels. This depolarisation opens the voltage-gated calcium channels and leads to unregulated insulin exocytosis. Although dominant acting *ABCC8/KCNJ11* mutations have been reported, recessively inherited mutations are more common [4, 5, 9, 10].

Diazoxide, which binds to the SUR1 subunit of the K_ATP_ channel and reduces insulin secretion by hyperpolarisation of the pancreatic β-cell plasma membrane, is the first line of treatment for CHI [1]. However, recessive inactivating mutations in *ABCC8* and *KCNJ11* usually cause severe diazoxide-unresponsive CHI due to defects in channel biogenesis, turnover, trafficking or regulation [11].

We describe a unique genotype-phenotype correlation with diazoxide responsive CHI in a patient with compound heterozygous *ABCC8* mutation. Functional work on the mutants was consistent with the observed clinical phenotype.

## Case presentation

### Clinical case

A term large-for-gestational age (birth weight 4500 g at 39 weeks gestation) female infant born to non-consanguineous Caucasian parents presented with symptomatic hypoglycaemia on first day of life. There was no history of gestational diabetes mellitus in the mother. The proband developed tonic clonic seizures associated with laboratory blood glucose of 0.4 mmol/l at 22 hours of age. The infection and metabolic screen was negative. She required infusion of high concentration glucose (glucose infusion rate 16 mg/kg/minute) to maintain blood glucose above 3.5 mmol/l.

A controlled hypoglycaemia screen established the diagnosis of CHI (serum Insulin 44.5 mU/l associated with lab glucose of 2.3 mmol/l and undetectable non-esterified fatty acids and β-hydroxybutyrate). Her serum cortisol was 570 nmol/l during the hypoglycaemia screen. The rest of the hypoglycaemic screen including insulin like growth factor-1 (IGF1), and insulin-like growth factor binding protein-3 (IGFBP3), serum ammonia, lactate, acylcarnitine profile, plasma amino acids and urine organic acids was within normal reference range (results not shown).

Molecular genetic analysis for CHI was performed after informed consent from the parents (see below). She was commenced on diazoxide (5 mg/kg/day in three divided doses) and the dose was gradually increased to 10 mg/kg/day. Chlorothiazide was given along with diazoxide to counteract the side effect of fluid retention. On 10 mg/kg/day of diazoxide, she was successfully weaned off intravenous glucose administration. She demonstrated age appropriate fasting tolerance on diazoxide before discharge.

Blood glucose monitoring performed at home demonstrated satisfactory glycaemic control on diazoxide. No adjustment in diazoxide dose was required with her growth. At 9 months of age, a trial off diazoxide therapy resulted in recurrence of hypoglycaemia with fasting tolerance of only 3½ hours. Diazoxide was recommenced at 5 mg/kg/day, which led to disappearance of hypoglycaemia and age appropriate fasting tolerance.

At the time of writing, the proband is 15 months old and is able to fast for 12 hours without developing hypoglycaemia on a low dose of diazoxide (5 mg/kg/day). Neurodevelopmental assessment did not identify any abnormality.

## Genetic analysis

### Methods

Genomic DNA was extracted from peripheral blood leukocytes using standard procedures. The single exon of the *KCNJ11* gene was amplified in 3 overlapping fragments and sequenced. When no mutations were identified in *KCNJ11*, the 39 exons of the *ABCC8* gene were amplified by polymerase chain reaction (PCR). The products were sequenced using Big Dye Terminator cycler sequencing Kit v3.1 (Applied Biosystems, Warrington, UK) and sequencing reactions were analysed on an ABI3730 (Applied Biosystems, Warrington, UK). Sequences were compared to the reference sequence (NM_000352.2) using Mutation Surveyor software (SoftGenetics, Pa., USA).

### Results

Sequence analysis identified biallelic *ABCC8* mutation in the proband – a missense mutation, R526C, inherited from an unaffected mother and a frameshift mutation, c.1879delC (H627Mfs*20), inherited from an unaffected father. Both R526C and c.1879delC (H627Mfs*20) mutations have previously been reported as recessive acting mutations in patients with focal CHI [5].

## Functional analysis of mutant channels

### Methods

Single point mutations (R526C and c.1879delC) were introduced into the hamster SUR1 clone by PCR using the Strategene XL Mutagenesis Kit according to the manufacturer’s instructions.

Human Embryonic Kidney (HEK) 293 cells were transiently transfected with Kir6.2/SUR1, Kir6.2/SUR1c1879delC, Kir6.2/SUR1R526C or Kir6.2/SUR1c1879C + SUR1R526C (together with a small amount of eGFP (Green Fluorescence Protein) expressing plasmid to enable identification of transfected cells using epifluorescence) using FuGENE HD (Roche Diagnostics, UK) as per the manufacturers’ instructions and cells were subjected to whole cell patch-clamp 48 hours after transfection.

Whole-cell patch-clamp recordings were performed as previously described [12]. Capacitance transients and series resistance in whole-cell recordings were compensated electronically by using amplifier circuitry (Multiclamp 700B). Data were filtered at 1 kHz using the filter provided with the Multiclamp 700B (4 pole Bessel) and sampled at 5 kHz using a Digidata 1440 (Axon Instruments). Currents were acquired and analysed using pClamp 10.4 (Axon Instruments). The intracellular (pipette) solution contained (mM); 140 KCl, 1.2 MgCl_2_, 1 CaCl_2_, 10 EGTA and 5 HEPES, 0.1 mM Na.ATP and 1 mM Na.ADP, pH 7.2 using KOH. The bath solution contained (mM); 5 KCl, 140 NaCl, 2.6 CaCl_2_, 1.2 MgCl_2_ and 5 HEPES (pH 7.4). Pipette resistances were between 2–4 mΩ. Diazoxide and Tolbutamide were obtained from Sigma Aldrich (Poole, UK). Agents were applied to the bath using a gravity-driven perfusion system.

### Results

Whole-cell patch-clamp recordings from HEK 293 cells transfected with wild-type (WT) Kir6.2/SUR1 cDNA showed normal K_ATP_ currents which was activated by the K_ATP_ channel opener diazoxide (100 μM) and inhibited by the K_ATP_ blocker tolbutamide (100 μM) (control, 144.77 ± 25.57 pA/pF; diazoxide, 382.7 ± 37.67 pA/pF; diazoxide + tolbutamide, 98.05 ± 28.05 pA/pF, n = 5 cells, P < 0.05). Currents from cells transfected with the frameshift mutation SUR1c1879delC were unresponsive to diazoxide and tolbutamide (control, 85.26 ± 12.7 pA/pF; diazoxide, 91.98 ± 14.36 pA/pF; tolbutamide, 79.57 ± 11.57 pA/pF, n = 7 cells, P > 0.05). In contrast, currents from cells transfected with the missense mutation, SUR1R526C, activated in the presence of diazoxide albeit to a lesser extent when compared to WT (control, 76.55 ± 8.2 pA/pF; diazoxide, 175.2 ± 16.47 pA/pF; tolbutamide, 64.55 ± 8.88 pA/pF, n = 5 cells, P < 0.05).

**Figure 1:**
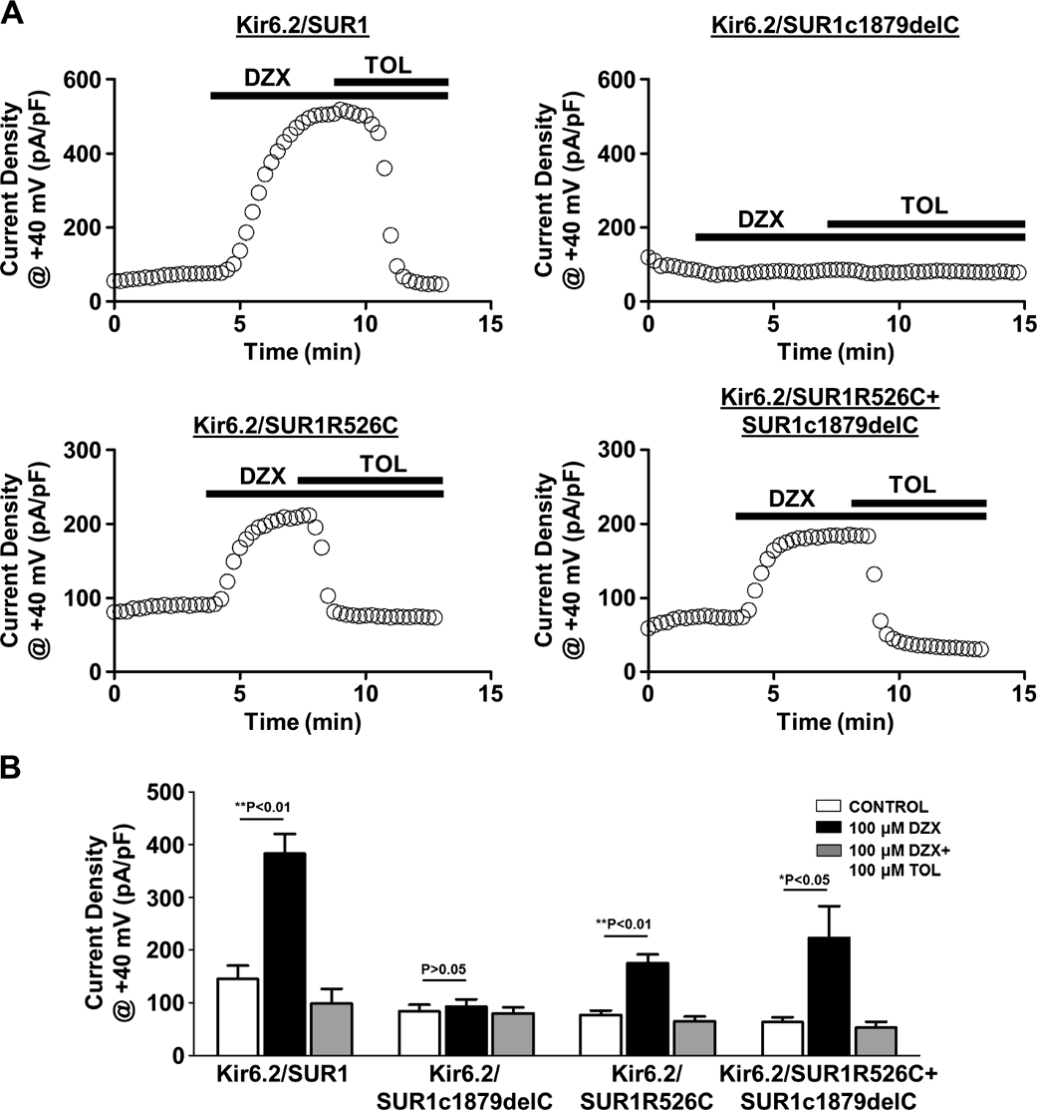
**Functional characterisation of K**_**ATP**_ **channels with a heterozygous** ***ABCC8*** R526C/H627Mfs*20 compound mutation. A, Representative whole-cell time-current density traces at +40 mV recorded from HEK293 cells expressing Kir6.2/SUR1, Kir6.2/SUR1c1879delC, Kir6.2/SUR1R526C and Kir6.2/SUR1R526C + SUR1C1879delC showing the effects of diazoxide (DZX) and tolbutamide (in the presence of diazoxide) (TOL). Current were recorded using a 1 s ramp protocol (-150 mV to 50 mV). **B**, Summary of the mean current-densities at +40 mV. Values are mean ± S.E.M from 5–7 cells, *P < 0.05, **P < 0.01 compared to control

Interestingly, co-expression of SUR1c1879delC and SUR1R526C (to mimic the compound heterozygous *ABCC8* (R256C/H627Mfs*20) mutation) rescued the diazoxide-sensitive K_ATP_ current which was absent when SUR1c1879delC was expressed alone (control, 62.9 ± 9.3 pA/pF; diazoxide, 223.11 ± 60.21 pA/pF; tolbutamide, 52.26 ± 11.36 pA/pF, n = 5 cells, P < 0.05). These data are shown in Figure 1. The diazoxide-sensitive K_ATP_ current in the double mutant transfected cells was significantly greater as compared to SUR1c1879delC transfected cells (160.2 ± 52.9 vs 8.72 ± 4.41 pA/pF, P = 0.01) and equivalent to WT Kir6.2/SUR1 transfected cells (160.2 ± 52.9 vs 237.9 ± 59.1 pA/pF, P = 0.33).

There was no statistically significant difference in the K_ATP_ current seen in cells transfected with SUR1R256C alone and cells co-transfected with SUR1R256C and SUR1c1879delC (p = 0.291).

## Discussion

We describe a patient with diazoxide responsive CHI due to compound heterozygous *ABCC8* mutation. The proband was macrosomic at birth (consistent with foetal hyperinsulinism) and presented with severe hypoketotic hypoglycaemia requiring high glucose infusion (16 mg/kg/minute). Investigations confirmed CHI and compound heterozygous *ABCC8* (R256C/H627Mfs*20) mutation. Surprisingly, the proband showed an excellent response to moderate doses of diazoxide (10 mg/kg/day). Subsequent follow-up revealed persistent requirement for diazoxide to control CHI. Functional analysis of the mutant K_ATP_ channel subunits confirmed a phenotype of diazoxide-responsive CHI in association with *ABCC8* R526C/H627Mfs*20 compound heterozygous mutation.

Mutations in *ABCC8* and *KCNJ11,* both monoallelic and biallelic, account for the majority of CHI patients [4, 5]. Although monoallelic *ABCC8/KCNJ11* mutations can cause both diazoxide-responsive as well as diazoxide-unresponsive CHI, nearly all biallelic *ABCC8/KCNJ11* mutations result in diazoxide unresponsive CHI [9, 10, 13, 14]. In two recent large studies comprising more than 700 patients with CHI, there was no patient reported with diazoxide responsive CHI due to biallelic *ABCC8/KCNJ11* mutation [4, 5].

However, Dekel et al. reported a patient with a compound heterozygous *ABCC8* mutation (c.3992-9G > A/F1388del) who responded to diazoxide [15]. However, no functional work was done to correlate with the clinical observations.

Previous functional work on compound heterozygous *ABCC8* mutations has shown that mutations may interact to modify K_ATP_ channel function and influence disease severity. Muzyamba et al. showed that single SUR1 mutants (D1193V or R1436Q) trafficked to the plasma membrane whereas the double mutant (SUR1D1193V/R1436Q) was retained in the endoplasmic reticulum [16].

Both SUR1 R526C and H627Mfs*20 mutations have been described previously as presumed recessive acting mutations in association with CHI. Snider et al. reported one patient each with both these mutations in association with focal CHI [5]. No functional work had been done on either of these mutations. The H627Mfs*20 mutation is a frameshift mutation and is severely damaging to the protein function as shown by our functional work. When the SUR1H627Mfs*20 mutant was co-expressed with Kir6.2, there was no increase in current with the application of diazoxide indicating either absence of K_ATP_ channels on the plasma membrane surface and\or severely dysfunctional K_ATP_ channel. With the SUR1R526C mutant, there was reduced current flow under basal conditions as compared to wild-type. However, importantly, there was an increase in current with the application of diazoxide which persisted when the two mutants (SUR1R526C/H627Mfs*20) were expressed in combination, suggesting this variant to be diazoxide-responsive.

As the frameshift mutation H627Mfs*20 results in a premature termination codon and is likely to be degraded by non-sense mediated decay, it is possible that the K_ATP_ channels in double mutant SUR1R256C/H627Mfs*20 will contain SUR1 subunits produced by allele carrying R526C mutation only and hence the response shown by the SUR1R256C/H627Mfs*20 resembles that of SUR1R256C mutant. However non-sense mediated decay cannot be reproduced in the expression studies as that requires replication of intronic and exonic structure of the gene whereas the plasmids can only accommodate cDNA of the gene.

Although we used 100 μM diazoxide to assess the responsiveness of SUR1 mutants, to our knowledge there is no data in the literature as to what concentration of diazoxide is achieved at the cellular level with the standard doses of diazoxide used for medical management of CHI. However, assuming a distribution volume of ~0.2 L/kg, the dose of diazoxide administered orally to this proband is likely to result in a concentration higher (~2×) than the 100 μM we used for our studies at the cellular level [17].

Our functional data, however, is not consistent with the previous observation of diazoxide-unresponsive focal CHI in association with paternally inherited heterozygous *ABCC8* R526C mutation [5]. Marked intrafamilial clinical heterogeneity in four haploidentical siblings harbouring the identical *ABCC8* homozygous c.3992-9G > A mutation was also highlighted by Kapoor et al. [18]. This difference in clinical expression may be due to background genetic factors and other unknown factors involved in regulating gene expression.

## Conclusion

In conclusion, although the majority of biallelic *ABCC8/KCNJ11* mutations result in diazoxide-unresponsive CHI, occasional biallelic and particularly compound heterozygous *ABCC8* mutations may lead to a diazoxide-responsive phenotype. Accurate clinical assessment would avoid the need for near-total pancreatectomy in such cases.

## Consent

Written informed consent was obtained from the patient for publication of this Case report and any accompanying images. A copy of the written consent is available for review by the Editor-in-Chief of this journal.

## Abbreviations

CHI: Congenital hyperinsulinism
KATP: Potassium sensitive ATP channel
SUR1: Sulfonylurea receptor 1
IGF1: Insulin-like growth factor 1
IGFBP3: IGF binding protein 3
PCR: Polymerase chain reaction
HEK 293 cells: Human Embryonic Kidney 293 cells
GFP: Green Fluorescence Protein
WT: Wild type.
